# A Closed-Loop Audit of Electronic Discharge Summary Completeness and Safety Netting at Queen Alia Military Hospital in Jordan

**DOI:** 10.7759/cureus.109079

**Published:** 2026-05-18

**Authors:** Murad M Hamiedah, Abdallah H Khamash, Ahmad Ibrahim Al-Arman, Omar J Haddad

**Affiliations:** 1 General Surgery, Jordanian Royal Medical Services, King Hussein Medical Center, Amman, JOR

**Keywords:** clinical audit system, discharge summary, documentation errors, hospital discharge, quality improvement and patient safety, surgery

## Abstract

Background

Safe discharge from the hospital is a critical component of patient safety and affects continuity of care. Following discharge, surgical patients remain at risk of developing some complications and may be uncertain about their expected recovery. Clear, structured discharge information will help them understand the post-discharge pathway, recognize warning symptoms, and seek timely medical review. Safety netting advice is, therefore, important and essential in reducing preventable harm and avoidable readmissions. This study aimed to enhance patient safety and continuity of care at Queen Alia Military Hospital (QAMH) by improving the quality of electronic surgical discharge summaries, with a specific focus on safety netting. The objectives included assessing baseline completeness of 100 summaries via a 12-parameter checklist; identifying clinical and systemic barriers through a resident survey; evaluating the impact of educational workshops and standardized templates via a 106-record re-audit; and determining the prevalence of safety-netting gaps regarding red-flag symptoms and pending histopathology results across both audit cycles.

Methodology

A prospective clinical audit loop was conducted by utilizing retrospective electronic record reviews for data collection at QAMH by evaluating the completeness of surgical discharge summaries using a 12-parameter checklist. Following a baseline audit of 100 consecutive discharges (December 2025 to January 2026), a multimodal intervention was implemented in February 2026, comprising resident educational workshops, standardized electronic templates, and dedicated documentation time. A resident survey (n = 15) identified systemic barriers, primarily time pressure and lack of training. A re-audit of 106 records (March 2026) was conducted to measure improvement. Data were analyzed via descriptive statistics and Fisher’s exact test, with inter-rater reliability ensured by independent specialist review.

Results

In a review of 100 adult surgical discharge summaries, overall completeness was moderate, with most summaries documenting only 5-7 out of 12 required items. Very few reached high completeness (8-9 items), and none included key safety-netting information such as red-flag symptoms, clear advice on when to seek help, or pending histopathology results. Statistical analysis showed that some sections tended to be completed together, such as final diagnosis with discharge condition, and procedures with discharge medications. However, other areas showed inconsistent or uneven documentation, with hospital course and physical assessment often missing when other sections were well documented, highlighting variability in how different parts of discharge summaries are completed. The re-audit demonstrated a substantial shift in documentation practices, with mean completeness rising to 77.04%, with core clinical reporting exceeding 90% compliance.

Conclusions

The implementation of clinical audit loops successfully standardized the reporting of objective clinical data, leading to a significant increase in the quality of discharge summaries. The dramatic rise in documented physical assessments and test results suggests that previous interventions effectively integrated these requirements into the clinical workflow. Nevertheless, a safety-netting gap persists. Future quality improvement cycles should prioritize the inclusion of patient-centered instructions.

## Introduction

Safe discharge from the hospital is essential to deliver high-quality surgical care and is an important step in ensuring patient safety and continuity of treatment. Discharge represents a major transition point, where responsibility for ongoing recovery shifts from hospital teams to patients, families, and primary care services. This period is recognized as particularly vulnerable, as breakdowns in communication or inadequate discharge planning can lead to medication errors, delayed follow-up, patient uncertainty, and avoidable harm [1].

Surgical patients are especially at risk during the post-discharge period because complications such as wound infection, bleeding, venous thromboembolism, or persistent pain may develop after leaving the hospital. Many patients may be unsure of what constitutes a normal recovery versus an early warning sign of deterioration. Without clear written guidance, patients may delay seeking help, potentially resulting in emergency readmissions or more serious outcomes [2].

Research indicates that up to 10-15% of surgical patients experience a complication within 30 days of discharge, with surgical-site infections being the most common, often manifesting 5-10 days post-surgery. Venous thromboembolism risk remains elevated for up to 90 days post-discharge, yet many patients lack the specific knowledge to identify early symptoms such as calf swelling or shortness of breath. Additionally, inadequate pain management and medication discrepancies affect up to 20% of surgical patients following discharge, often leading to unnecessary emergency department visits [3-5].

The discharge summary plays a central role in supporting safe transitions of care. It provides essential clinical information to general practitioners and community services, including details of diagnoses, procedures performed, medication changes, follow-up arrangements, and patient-specific instructions. However, studies have consistently shown that discharge summaries are often incomplete, delayed, or variable in quality, which can compromise ongoing care in the community [1].

In addition to clinical handovers, patient-facing safety netting advice is increasingly recognized as a key element of safe discharge practice. Safety netting involves providing patients with clear instructions about expected recovery, symptoms that should prompt urgent medical attention, and how to access appropriate support. Almond et al. highlighted safety netting as an important strategy to reduce preventable harm, particularly when complications may evolve after discharge [6].

National standards reinforce the importance of structured discharge documentation. In the United Kingdom, the Professional Record Standards Body sets out recommended content for discharge summaries, emphasizing the inclusion of management plans, medication changes, and advice for patients following discharge [7]. Similarly, the General Medical Council stresses the professional duty of clinicians to ensure safe handover and effective communication when transferring care responsibility [8].

McMillan et al. (2006) demonstrated that hospital discharge summaries often contain significant inaccuracies, compromise patient safety during the transition of care, with a potential to cause serious consequences [9]. Insufficient documentation in surgical discharge summaries is a pervasive issue, with studies showing that up to one-third of summaries in the United States and 40% in Europe miss crucial clinical details required for safe transitions of care [10].

Given the critical role of discharge summaries as vital educational and safety tools, this study addresses frequently missed opportunities in documentation to ensure high-quality continuity of care during the transition from hospital to home. Therefore, the primary research question guiding this study is how does the implementation of a multi-modal educational intervention and standardized templates impact the completeness of surgical discharge summaries, particularly regarding safety-netting advice? To answer this question, the specific objectives of this closed-loop clinical audit are to (1) assess the baseline completeness of electronic surgical discharge summaries using a 12-parameter checklist; (2) identify clinical and systemic barriers to documentation through a resident clinician survey; (3) evaluate the impact of educational workshops and standardized templates via a subsequent re-audit; and (4) determine the prevalence of safety-netting gaps concerning red-flag symptoms and pending histopathology results across both audit cycles [11].

## Materials and methods

This quality improvement study was designed, conducted, and reported in accordance with the Revised Standards for Quality Improvement Reporting Excellence (SQUIRE 2.0) guidelines. This study was designed as a prospective clinical audit loop utilizing retrospective electronic record reviews to evaluate and improve the quality of surgical discharge summaries. The audit was conducted in the Surgical Department of Queen Alia Military Hospital (QAMH), a 100-bed surgical facility within the Jordanian Royal Medical Services.

Sampling and population

A convenience sampling method was used for both cycles, targeting the department’s total monthly throughput. The baseline audit included 100 consecutive adult surgical discharges (≥18 years) between December 1, 2025, and January 1, 2026. Following the intervention phase, a re-audit of 106 consecutive records was performed between March 1 and March 31, 2026. Pediatric patients and those who were discharged against medical advice were excluded to ensure a standardized focus on routine clinical transitions. The rationale for excluding pediatric patients was that their discharge process involves distinct communication dynamics with parents or guardians, weight-based pharmacological dosing, and specialized pediatric early-warning criteria that require a different standardized template and safety-netting approach compared to adult surgical patients.

Data collection and reliability completeness were measured against 12 standardized parameters established by the Jordanian Royal Medical Services. To ensure inter-rater reliability and minimize bias, two independent surgical specialists reviewed each summary using a binary (Yes/No) checklist. Any discrepancies in documentation assessment were resolved through adjudication by a third senior consultant.

Intervention phase

Between the two audit cycles (February 1 to February 28, 2026), a multimodal educational intervention was led by the authors and implemented by the surgical specialist teams. This consisted of teaching sessions, seminars, and training on a standardized discharge summary so they are familiar with it, putting visual reminders on the electronic system, and sessions on the importance of documentation and its legal issues.

To identify systemic barriers, a 10-item questionnaire, which was developed by the authors, was administered to 15 surgical residents. The survey instrument underwent content validation via a pilot review by three senior surgical consultants to ensure the questions effectively captured issues regarding workload and training. There were no selection criteria for the surgical participants, as all 15 surgical residents participated in the questionnaire, currently rotating through the general surgery subspecialties (hepatobiliary, colorectal, bariatric, and oncology).

Data were analyzed using descriptive statistics to calculate mean completeness. Fisher’s exact test was employed to identify associations between documented clinical parameters. This audit received formal approval from the Ethical Committee of the Jordanian Royal Medical Services.

Standards

The standards are local discharge summary standards developed by the Jordanian Royal Medical Services, and they include the following parameters: history and reason for admission, physical assessment at discharge, procedures and treatment done during admission, significant results, hospital course, final diagnosis, condition upon discharge, outpatient medication, follow-up plan, red flags and when to seek help, pending histopathology results, and actionable safety netting (Table 1).

**Table 1:** The 12 local parameters standards at the Jordanian Royal Medical Services.

	Parameter
1	History/Reason for admission
2	Physical assessment
3	Procedures and treatment
4	Significant results
5	Hospital course
6	Final diagnosis
7	Condition on discharge
8	Outpatient medications
9	Follow-up plan (date & location)
10	Red flags/When to seek help
11	Pending histopathology results
12	Actionable safety netting

## Results

Across 100 adult surgical discharge summaries, completeness was quantified as the number of documented items across 12 parameters (Yes = 1/No = 0). Scores ranged from 2 to 9 (mean = 5.81 ± 1.39; median = 6, interquartile range (IQR) = 5-7), with the distribution clustered at 5-7 items (77% of summaries) and only 8% achieving 8-9 items (Tables 2, 3).

**Table 2:** Parameter completeness (Yes/No) and their percentages.

	Parameter	Yes (n)	No (n)	Yes (%)
1	History/Reason for admission	96	4	96.0%
2	Physical assessment	36	64	36.0%
3	Procedures and treatment	69	31	69.0%
4	Significant results	27	73	27.0%
5	Hospital course	88	12	88.0%
6	Final diagnosis	51	49	51.0%
7	Condition on discharge	64	36	64.0%
8	Outpatient medications	61	39	61.0%
9	Follow-up plan (date and location)	89	11	89.0%
10	Red flags/When to seek help	0	100	0.0%
11	Pending histopathology results	0	100	0.0%
12	Actionable safety netting	0	100	0.0%

**Table 3:** Score distribution (number of checklist items documented as Yes).

Score	Count	Percent
0	0	0.0%
1	0	0.0%
2	1	1.0%
3	3	3.0%
4	11	11.0%
5	32	32.0%
6	16	16.0%
7	29	29.0%
8	5	5.0%
9	3	3.0%
10	0	0.0%
11	0	0.0%
12	0	0.0%

The maximum observed score was 9/12 because three safety-netting-related parameters (red-flag symptoms/when to seek help, actionable safety-netting advice, and anticipated histopathology/pending results) were absent in all discharge summaries (0% compliance). This means the “typical” discharge summary in this audit contained about half of the expected elements (median = 6/12), and the absence of safety‑netting/pending‑results documentation was a system‑wide gap, not an occasional omission (Tables 4, 5).

**Table 4:** Overall completeness of discharge summaries.

Overall completeness	
Mean score (/12)	5.8
Median score (/12)	6.0
Minimum–maximum (/12)	2–9
Mean completeness (%)	48.4%

**Table 5:** Age distribution in years. Number of patients (n = 100). Adults included (≥18).

Age (years)	
Mean	45.5
SD	18.8
Median	44.0
Minimum	18
Maximum	90

Analysis of documentation patterns using Fisher’s exact test demonstrated a strong positive association between documentation of final diagnosis and condition on discharge (odds ratio (OR) = 5.73, 95% confidence interval (CI) = 2.30-14.28; p = 0.00014) and between procedures/treatment and discharge medications (OR = 3.88, 95% CI = 1.59-9.45; p = 0.0036). In contrast, significant results were inversely associated with documentation of hospital course (OR = 0.086, 95% CI = 0.021-0.350; p = 0.00030), and physical assessment was inversely associated with final diagnosis documentation (OR = 0.216, 95% CI = 0.089-0.526; p = 0.00076), suggesting heterogeneous completion patterns across sections of the discharge summary.

The re-audit on 106 surgical discharge summaries revealed remarkable improvements in nearly all categories compared to the baseline. Documentation of physical assessments increased from 36.0% to 98.1%, while the inclusion of significant results increased from 27.0% to 92.5%. High-level compliance (>90%) was maintained for history (100%), follow-up plans (99.1%), and outpatient medications (96.2%). Notably, parameters that were entirely absent in the initial audit showed emerging compliance: safety netting increased to 33.0%, and red-flag symptom documentation reached 17.9%. However, documentation of anticipated histopathology results remained the lowest-performing metric at 3.8% (Table 6).

**Table 6:** Percentage of discharge summaries that correctly included each clinical parameter during the audit cycle.

Audit parameter	Original audit (%)	Re-audit (%)	Improvement (%)
History and reason for admission	96.00%	100.00%	+4.0%
Physical assessment	36.00%	98.10%	+62.1%
Procedures and treatment	69.00%	95.30%	+26.3%
Significant results	27.00%	92.50%	+65.5%
Hospital course	88.00%	96.20%	+8.2%
Final diagnosis	51.00%	94.30%	+43.3%
Condition upon discharge	64.00%	98.10%	+34.1%
Outpatient medications	61.00%	96.20%	+35.2%
Follow-up plan (date and location)	89.00%	99.10%	+10.1%
Red-flag symptoms/Safety netting	0.00%	17.90%	+17.9%
Actionable safety netting	0.00%	33.00%	+33.0%
Information on pending histopathology	0.00%	3.80%	+3.8%

The comparative analysis of 100 original and 106 re-audit summaries demonstrated a statistically significant improvement across 10 of the 12 parameters (p < 0.05). The mean completeness score increased from 5.81 ± 1.39 to 9.24 ± 0.84 (p < 0.001), with the most profound gains in physical assessment (36% to 98.1%, p < 0.001) and significant results (27% to 92.5%, p < 0.001). Core clinical data, such as final diagnosis and outpatient medications, also reached high-level compliance (>94%, p < 0.001). While safety-netting elements, including red-flag symptoms and actionable advice, showed significant growth from a zero baseline (p < 0.001), they remained the lowest-performing areas. Documentation of pending histopathology results demonstrated a marginal increase from 0% in the baseline audit to 3.8% in the re-audit; however, this did not reach statistical significance (p = 0.057).

**Figure 1:**
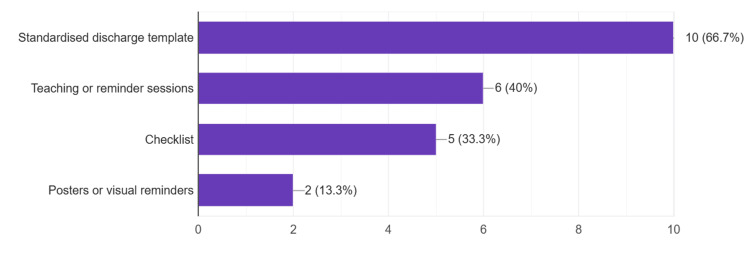
Results based on a questionnaire collected from 15 surgical residents regarding how to improve the documentation of discharge summaries.

**Figure 2:**
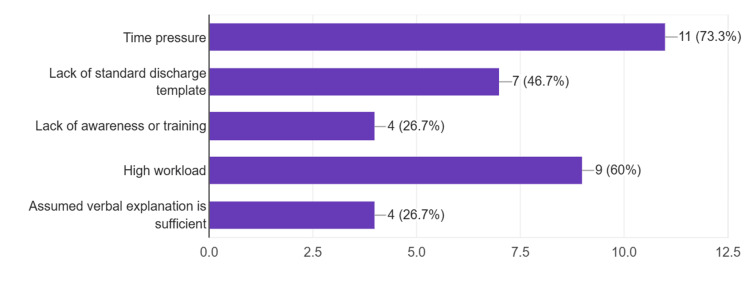
Results based on a questionnaire collected from 15 surgical residents regarding the main barriers that prevent them from completing the discharge summaries.

The survey of 15 resident clinicians revealed that while 80% (n = 12) were aware of the importance of documenting safety-netting advice, actual practice was inconsistent, with only 40% (n = 6) reporting they “always” routinely document it. The primary barriers identified were time pressure (cited by 87%, n = 13) and high workload (60%, n = 9), alongside a lack of standardized templates and formal training; notably, 73% (n = 11) of respondents reported receiving no formal training on the subject. To improve documentation quality, clinicians most frequently suggested implementing a standardized discharge template (60%, n = 9) and providing a dedicated checklist (40%, n = 6) as the most effective interventions (Figures 1, 2).

## Discussion

This audit demonstrates that the overall completeness of surgical discharge summaries in our unit was moderate, with most summaries documenting only around half of the expected elements. The majority of discharge summaries were clustered between 5 and 7 documented items, and only a small proportion achieved high completeness. This finding is consistent with previous studies showing that discharge summaries are frequently incomplete and variable in quality, despite their central role in ensuring safe transitions of care [1,2]. Mikkelsen et al. (2024) found that nearly half of all discharge summaries fail to provide clear, actionable follow-up instructions for general practitioners, emphasizing that the inclusion of dedicated, high-visibility recommendation fields is critical for reducing patient risk and preventing avoidable readmissions [12].

A key finding of this audit is the complete absence of safety-netting documentation, including red-flag symptoms, advice on when and where to seek help, and documentation of pending histopathology results. This represents a system-wide gap rather than isolated individual omissions. Similar deficiencies have been reported in the literature, where discharge summaries often prioritize inpatient clinical information while neglecting patient-focused guidance for post-discharge care [1]. Given that surgical complications frequently develop after discharge, the lack of clear written safety-netting advice may place patients at increased risk of delayed presentation and avoidable readmissions [2].

While the intervention yielded substantial, statistically significant improvements in objective clinical parameters, most notably the increase in physical assessment documentation from 36% to 98.1%, the impact on safety-netting was comparatively subdued. Although documentation of red-flag symptoms and general safety netting improved, they only reached 17.9% and 33%, respectively, in the re-audit. We acknowledge that these figures remain critically below acceptable clinical standards. This discrepancy likely stems from the fundamental nature of the documentation. Objective parameters can be rapidly satisfied using rigid checklists or standardized templates. In contrast, robust safety netting, particularly for complex general surgery, HPB, or trauma discharges, requires highly individualized, patient-specific advice that cannot be easily automated. High cognitive load, time constraints during busy ward rounds, and the frequent delegation of these summaries to junior staff likely contribute to why these crucial, personalized parameters showed the least improvement.

The observed associations between certain documentation elements suggest that clinicians tend to complete discharge summaries in thematic clusters. For example, the final diagnosis was strongly associated with documentation of the condition on discharge, and procedures were frequently documented alongside discharge medications. Similar patterns have been described in previous studies and may reflect clinician workflow, time pressures, or the structure of electronic discharge templates [2]. In contrast, the inconsistent documentation of physical assessment, significant results, and hospital course highlights variability in clinical practice and suggests that some sections are perceived as lower priority.

The importance of safety netting has been well described by Almond et al., who emphasized that clear guidance regarding warning symptoms and follow-up actions is essential in reducing preventable harm, particularly in situations of diagnostic uncertainty [6]. This is especially relevant in surgical patients, where postoperative complications such as wound infection, bleeding, or venous thromboembolism may evolve after discharge and may not be immediately recognized by patients.

National standards emphasize that discharge summaries should support both clinician-to-clinician communication and patient understanding. The Professional Record Standards Body recommends inclusion of clear diagnoses, management plans, medication changes, follow-up arrangements, and patient advice [7], while the General Medical Council highlights the professional duty to ensure safe handover and effective communication at transitions of care [8]. The findings of this audit suggest that current practice does not fully meet these standards, particularly in relation to patient-centered safety-netting information.

Limitations

This study has several notable limitations. First, the reliance on convenience sampling from a single military center (QAMH) may restrict the generalizability of our findings to other hospitals with different resource allocations. Second, the resident survey utilized a small sample size (n = 15), which may not fully capture the diverse systemic barriers experienced by different doctors in other hospitals. Third, this audit focused strictly on documentation completeness and did not evaluate patient-level outcomes, such as the impact of these enhanced summaries on 30-day readmission rates, complication rates, or patient satisfaction. Finally, while the re-audit demonstrated immediate, statistically significant improvements, it was conducted shortly after the intervention phase due to logistics; therefore, further longitudinal evaluation is required to assess the long-term sustainability of these improved documentation practices.

## Conclusions

This audit identified moderate-quality surgical discharge summaries characterized by a critical lack of patient-focused elements, such as safety-netting advice, red-flag symptoms, and pending results, a system-wide deficiency that heightens the risk of delayed complication recognition and avoidable readmissions. While the implementation of multimodal educational interventions and standardized templates successfully optimized routine clinical documentation, these standard approaches proved insufficient for more complex, personalized safety parameters. Consequently, future quality improvement cycles must transition beyond basic documentation standards toward targeted, system-level prompts and senior-led education to bridge the gap in safety netting, complemented by regular re-auditing to embed these high-quality, patient-centric practices within the surgical department’s culture.

## Appendices

**Table 7:** Questionnaire developed by the authors for this audit to evaluate the limitations of the completeness of discharge summaries.

What is your current role?	Are you aware of the importance of documenting safety netting advice in discharge?	Do you routinely document safety-netting advice when completing discharge summaries?	What are the main barriers to documenting adequate safety-netting advice?	How confident are you in knowing what constitutes adequate safety-netting advice?	What would help improve safety-netting documentation?	When do you usually complete discharge summaries?	Have you received any formal training on discharge safety-netting documentation?	Do you complete all mandatory sections of the discharge summary template?
Resident	Somewhat	Rarely	Time pressure, lack of standard discharge template, high workload, assumed verbal explanation is sufficient	Somewhat confident	Checklist	During working hours	No	Yes
Resident	Yes	Always	Time pressure	Very confident	Teaching or reminder sessions	After patient discharge	Yes	No
Resident	Yes	Always	High workload	Very confident	Posters or visual reminders	Variable	Yes	Yes
Resident	Yes	Sometimes	Time pressure, lack of standard discharge template, lack of awareness or training, high workload	Somewhat confident	Standardized discharge template, teaching or reminder sessions, checklist, posters or visual reminders	During working hours	No	No
Resident	Yes	Always	Time pressure, lack of standard discharge template, high workload	Somewhat confident	Standardized discharge template	During working hours	No	Partially complete
Resident	Yes	Sometimes	Lack of standard discharge template, lack of awareness or training, high workload, assumed verbal explanation is sufficient	Somewhat confident	Standardized discharge template, teaching or reminder sessions	During working hours	No	Yes
Resident	Yes	Rarely	Lack of a standard discharge template, high workload, assumed verbal explanation is sufficient	Somewhat confident	Standardized discharge template, teaching or reminder sessions, checklist	During working hours	No	Partially complete
Resident	Yes	Always	Time pressure	Somewhat confident	Standardized discharge template	During working hours	Yes	Yes
Resident	Yes	Always	Time pressure	Very confident	Standardized discharge template	During working hours	Yes	Yes
Resident	Yes	Often	Lack of awareness or training	Somewhat confident	Teaching or reminder sessions	During working hours	Yes	Yes
Resident	Yes	Always	Time pressure, lack of a standard discharge template, high workload	Somewhat confident	Standardized discharge template	During working hours	No	Partially complete
Resident	Somewhat	Sometimes	Time pressure, lack of standard discharge template, lack of awareness or training	Somewhat confident	Standardized discharge template, teaching or reminder sessions	During working hours	Yes	Partially complete
Resident	Yes	Often	Time pressure, high workload, assumed verbal explanation is sufficient	Very confident	Standardized discharge template	During working hours	No	Partially complete
Resident	Yes	Always	Time pressure	Very confident	Checklist	During working hours	No	Yes
Resident	Yes	Often	Time pressure, high workload	Somewhat confident	Standardized discharge template, checklist	During working hours	No	Partially complete
